# Altered Variability and Concordance of Dynamic Resting-State Functional Magnetic Resonance Imaging Indices in Patients With Major Depressive Disorder and Childhood Trauma

**DOI:** 10.3389/fnins.2022.852799

**Published:** 2022-05-09

**Authors:** Qianyi Luo, Huiwen Yu, Juran Chen, Xinyi Lin, Zhiyao Wu, Jiazheng Yao, Yuhong Li, Huawang Wu, Hongjun Peng

**Affiliations:** ^1^Department of Clinical Psychology, The Affiliated Brain Hospital of Guangzhou Medical University, Guangzhou, China; ^2^Department of Radiology, The Affiliated Brain Hospital of Guangzhou Medical University, Guangzhou, China

**Keywords:** major depressive disorder, childhood trauma, resting-state functional magnetic resonance imaging, concordance, temporal dynamics

## Abstract

Childhood trauma is a non-specific risk factor for major depressive disorder (MDD). resting-state functional magnetic resonance imaging (R-fMRI) studies have demonstrated changes in regional brain activity in patients with MDD who experienced childhood trauma. However, previous studies have mainly focused on static characteristics of regional brain activity. This study aimed to determine the specific brain regions associated with MDD with childhood trauma by performing temporal dynamic analysis of R-fMRI data in three groups of patients: patients with childhood trauma-associated MDD (*n* = 48), patients without childhood trauma-associated MDD (*n* = 30), and healthy controls (*n* = 103). Dynamics and concordance of R-fMRI indices were calculated and analyzed. In patients with childhood trauma-associated MDD, a lower dynamic amplitude of low-frequency fluctuations was found in the left lingual gyrus, whereas a lower dynamic degree of centrality was observed in the right lingual gyrus and right calcarine cortex. Patients with childhood trauma-associated MDD showed a lower voxel-wise concordance in the left middle temporal and bilateral calcarine cortices. Moreover, group differences (depressed or not) significantly moderated the relationship between voxel-wise concordance in the right calcarine cortex and childhood trauma history. Overall, patients with childhood trauma-associated MDD demonstrated aberrant variability and concordance in intrinsic brain activity. These aberrances may be an underlying neurobiological mechanism that explains MDD from the perspective of temporal dynamics.

## Introduction

Major depressive disorder (MDD) is a common mental illness that affects over 350 million people. It is a heterogeneous clinical syndrome that can include symptoms of disturbed mood, difficulty concentrating, bodily complaints, self-loathing, delusions of guilt, indecision, and even a strong wish to die (de Kwaasteniet et al., 2013; McCarron et al., 2021). MDD is a major leading cause of disability and has an approximate 12-month prevalence of 6% worldwide (Kessler and Bromet, 2013). The onset and development of MDD is a complicated process and involves various factors, including genetic vulnerability (Howard et al., 2019), stressful life events and circumstances (Hammen, 2005; Southwick et al., 2005), dysfunctional cognition (Gotlib and Joormann, 2010; Figueroa et al., 2015), interpersonal dysfunction (Hammen and Brennan, 2002), female sex (Kuehner, 2017; Salk et al., 2017), and childhood trauma (Huh et al., 2017; Nelson et al., 2017).

Childhood trauma is a non-specific risk factor for MDD. Patients with childhood trauma-associated MDD have a worse treatment response (Nikkheslat et al., 2020). According to existing studies, among individuals with childhood trauma, 54% suffer from depression, 64% are addicted to illicit drugs, and 67% have experienced suicidal ideation (Dube et al., 2003). Childhood trauma consists of emotional, physical, and sexual abuse, and emotional and physical neglect (Danese and Baldwin, 2017), and has been closely associated with numerous psychiatric disorders such as MDD (Yu M. et al., 2019), bipolar disorder (Begemann et al., 2021), post-traumatic stress disorder (Kisely et al., 2018), and borderline personality disorder (Nicol et al., 2015). The neurobiological mechanisms underlying the association remain unclear. Yu M. et al. (2019) found that traumatic childhood experiences and dimensional symptoms are linked to aberrant network architecture in MDD, providing strong evidence for the negative impact of childhood trauma. Furthermore, Heim et al. (2008) and Du et al. (2016), observed an aberrant amplitude of low-frequency fluctuation (ALFF) and fractional amplitude of low-frequency fluctuation (fALFF) in patients with MDD across widespread brain regions relative to healthy controls, demonstrating that childhood trauma might lead to brain dysfunction and increased risk of MDD. Similarly, in a multimodal study, Duncan et al. (2015) found that childhood trauma causes long-term functional and structural effects in the brain.

Although previous studies have provided insights into the neurobiological mechanisms underlying MDD in patients who experienced childhood trauma, they did not examine variability and concordance in intrinsic brain activity. Brain activity fluctuates and changes over time in response to context and activity and underlies temporal-dynamic integration in the brain (Park et al., 2018). A number of studies have captured the temporal dynamic patterns of intrinsic brain activity using the sliding window method. Evidence has indicated that aberrant variability and concordance of resting-state functional magnetic resonance imaging (R-fMRI) indices are related to the mechanisms underlying MDD (Hutchison et al., 2013; Allen et al., 2014; Xue et al., 2020). Regarding aberrant variability, Zhao, Lei and colleagues reported significantly decreased dynamic ALFF (dALFF) in the emotion network in depressed patients (Zhao et al., 2021). Xue et al. (2020) observed a consistently decreased dynamic regional homogeneity (dReHo) in patients with MDD in both fusiform gyri, the right temporal pole, and the hippocampus relative to healthy controls. Additionally, Zhang et al. (2022) revealed the relationship between brain dynamic working patterns and chronic stress in adolescent MDD using the dynamic functional connectivity (FC) method. Zhu et al. (2020) reported abnormal cerebellar-cerebral dynamic FC changes in MDD. As for abnormal concordance, Zhu et al. (2019) reported decreased volume-wise concordance in patients with MDD relative to healthy controls. To characterize the local characteristics of the single voxel, ALFF and its normalized version fALFF have been used to compute the mean value of amplitudes within the 0.01–0.1 Hz low-frequency range from a Fourier decomposition of the blood oxygenation level-dependent (BOLD) time course (Zang et al., 2007; Zou et al., 2008). Regional homogeneity (ReHo) was developed to represent the level of regional brain activity coherence (Zang et al., 2004). Voxel-mirrored homotopic connectivity (VMHC) was adopted as the Pearson’s correlation coefficient between the time series of each voxel in one hemisphere and the time series of its symmetrical counterpart in the opposite hemisphere (Zuo et al., 2010b). Global signal connectivity (GSCorr) was considered as the Pearson’s correlation coefficient between the averaged time series and the time series of each voxel within the entire gray matter (Hahamy et al., 2014; Yang et al., 2017; Zhang et al., 2019). To depict the functional importance of the specific voxel, degree centrality (DC) was developed to calculate FC within the whole brain using the graph-theoretical approach (Buckner et al., 2009; Tomasi and Volkow, 2010; Zuo et al., 2012; Liu et al., 2015). Collectively, those R-fMRI indices have been applied widely to investigate aberrant intrinsic brain activity in depressed patients, which has enabled significant breakthroughs in the exploration of MDD neurobiological mechanisms (Guo et al., 2012; Liu et al., 2013, 2014; Shen et al., 2015; Gong et al., 2020; Ebneabbasi et al., 2021; Zhou et al., 2021). Therefore, in this study, we extensively applied dALFF, dynamic fALFF (dfALFF), dReHo, dynamic voxel mirrored homotopic connectivity (dVMHC), dynamic global signal correlation (dGSCorr), and dynamic DC (dDC) to investigate functional alterations of the brain in patients with MDD who experienced childhood trauma.

Previous studies have not explored alterations in variability and concordance of brain activity in MDD with childhood trauma. This study compared temporal dynamics analysis data based on R-fMRI images acquired from patients with MDD who experienced childhood trauma with data from patients with MDD who did not experience childhood trauma as well as healthy controls. We hypothesized that patients with MDD who experienced childhood trauma exhibit aberrant dynamic regional brain activity and concordance and that the concordance is associated with the severity of childhood trauma.

## Materials and Methods

### Participants

We recruited 78 patients with MDD and 108 healthy subjects for this study. MDD diagnosis was made by two psychiatrists with extensive experience using the DSM-5 diagnostic criteria. We used the Hamilton Depressive Rating Scale (HAMD) (Helmreich et al., 2012) to assess the depression severity (for those with MDD). It is well established that the Childhood Trauma Questionnaire (CTQ) is a reliable tool to evaluate the negative influence of maltreatment experience (Wu et al., 2022). Prior studies have proved that the CTQ has high validity in different countries (Kim et al., 2013; Isvoranu et al., 2017; Zhang et al., 2020; Petrikova et al., 2021). Using the CTQ cutoff points for the CTQ subscale scores to determine whether participants with and without traumatic experience has been widely validated and accepted (Jansen et al., 2016; Xie et al., 2018; Monteleone et al., 2020). Hence, we followed the same criterion to identify whether the participants suffered childhood maltreatment. To summarize, we used the cutoff point for the CTQ subscales score to distinguish the participants with and without childhood trauma, and the CTQ total score was used to quantify the severity of childhood maltreatment history. The CTQ total score and its subscale scores were used as continuous variables in this study. According to different types of childhood maltreatment, the CTQ can be divided into the following subscales: (i) emotional neglect (EN), (ii) physical neglect (PN), (iii) emotional abuse (EA), (iv) physical abuse (PA), and (vi) sexual abuse (SA) (Xie et al., 2018). The detailed cutoff points of CTQ subscales are shown below: (i) EN score ≥ 15, (ii) PN score ≥ 10, (iii) EA score ≥ 13, (iv) PA score ≥ 10, and SA score ≥ 8 (Jansen et al., 2016; Xie et al., 2018). Participants with any above-threshold score in the childhood trauma subtype will be considered as exposed to childhood maltreatment and will be included in our study. All participants received an assessment of the negative impact of traumatic history.

Based on whether each participant with or without traumatic history (using the cutoff points of the CTQ subscales to identify the participants with trauma exposure), had or had not been diagnosed with MDD (diagnosis of the patient with MDD was made by two psychiatrists), participants were divided into MDD with childhood trauma group (*n* = 48), MDD without childhood trauma group (*n* = 30), and healthy control group (*n* = 108). Patients with MDD were recruited from the inpatient department of the Affiliated Brain Hospital of Guangzhou Medical University. Correspondingly, 108 age-, gender-, and education-matched healthy controls were recruited from the advertising and nearby community. We excluded patients who (i) did not have a first episode of depression, (ii) had a history of any other major mental illness and physical disorder, (iii) had a family history of any other major mental illness and physical disorder, (iv) were taking psychiatric medication before (non-drug-naive), (v) received systemic psychotherapy and electroconvulsive therapy before, and (vi) were with contraindication for R-fMRI. The study was approved by the Ethics Committee of the Affiliated Brain Hospital of Guangzhou Medical University. All participants offered their written informed consent before the data collection.

### Magnetic Resonance Imaging Data Acquisition

MRI images were obtained using a 3T Philips scanner at the radiology department of The Affiliated Brain Hospital of Guangzhou Medical University in China. (i) Resting-state functional scans were performed using a gradient-echo echoplanar imaging sequence with the parameters listed below: TR = 2,000 ms, TE = 30 ms, number of slices = 33, flip angle = 90^°^, matrix = 64 × 64, field of view = 220 × 220 mm^2^, and slice thickness = 4 mm with 0.6 mm interslice gap. The whole scanning process included 240 time points, lasting for 8 min. (ii) High-resolution 3D T1 images were acquired with the parameters listed below: TR/TE = 8.2/3.7 ms, number of slices = 188, slice thickness = 1 mm, flip angle = 7^°^, acquisition matrix = 256 × 256, and voxel size = 1 mm × 1 mm × 1 mm. All participants were instructed to close their eyes, relax, remain motionless, and keep awake.

### Magnetic Resonance Imaging Data Preprocessing

Using the DPARSF toolbox (DPARSFA^1^) to preprocess the R-fMRI images, first, we removed the first ten volumes to allow data to reach equilibrium; second, we performed slice timing and head motion. Notably, the mean framewise displacement (FD) based on the Jenkinson model (FD-Jenkinson) was computed by averaging the FD from every time point for each subject (Jenkinson et al., 2002). We included only subjects with relatively low head motion (criteria: mean FD < 0.2 mm). Third, we conducted structural image alignment with a six-degree-of-freedom linear transformation to align the T1 image to the functional image; subsequently, we segmented the transformed structural images into the cerebrospinal fluid, white matter, and gray matter (Ashburner and Friston, 2005); Then, we spatially normalized the motion-corrected functional images into standard MNI space with 3 mm × 3 mm × 3 mm using the normalization parameters estimated during unified segmentation. The normalized images of the resulting ALFF and fALFF were then smoothed using a 4-mm FWHM Gaussian kernel. Subsequently, we treated Friston 24-head motion parameters, the white matter signal, and the CSF signal as the nuisance covariates to regress out (Friston et al., 1996). As for calculating ReHo, VMHC, and DC, the normalized images were subjected to nuisance regression to regress out Friston 24-head motion parameters, the white matter signal, and the CSF signal (Friston et al., 1996). Finally, the images were filtered with a temporal band-pass filter between 0.01 and 0.08 Hz.

### Dynamic Resting-State Functional Magnetic Resonance Imaging Indices Calculation

Dynamic indices were computed using the temporal dynamic analysis toolkits on DPABI (Yan et al., 2016) (DPABI,^2^ version 4.5). We used the sliding-window approach to explore alterations in variability and concordance of dynamic R-fMRI indices throughout the whole brain. For the calculation of the dynamic R-fMRI indices, window length is an essential but open parameter. Prior research has pointed out that 50 TRs window length is the most suitable parameter to maintain the balance between achieving reliable estimates of intrinsic brain activity (with a longer window length) and capturing high-speed shifting dynamic brain activity (with a shorter window length) (Liao et al., 2019; Cui et al., 2020; Liu et al., 2021). Thus, a sliding window length of 50 TRs and a step size of 1 TR were selected to analyze the dynamic R-fMRI indices in this study.

The time series of each subject was divided into 181 windows. In each window, R-fMRI metrics, including ALFF, fALFF, ReHo, GSCorr, VMHC, and DC, were calculated. Then, the following dynamic indices were analyzed: dALFF, dfALFF, dReHo, dGSCorr, dVMHC, and dDC. Images for calculating ALFF and fALFF were smoothed but not filtered; the images for calculating the other indices were filtered but not smoothed. A standard deviation (SD) across the windows was then computed to represent the dynamic indices. Finally, smoothing and Z standardization were executed on the SD maps (apart from dALFF and dfALFF, which were smoothed before). Window sizes of 30 TRs and 70 TRs were also computed (refer to Supplementary Data).

### Computation of Multiple Resting-State Functional Magnetic Resonance Imaging Indices

Interdependence among the following six R-fMRI brain activity indices was investigated:

(i) ALFF and fALFF: Above all, we transformed the time course into the frequency domain to acquire the corresponding power spectrum by using a Fast Fourier Transform. Then, we computed the square root at each frequency of the power spectrum. In particular, the averaged square root within the 0.01–0.1 Hz low-frequency range was considered the ALFF value (Zang et al., 2007). Moreover, fALFF was accepted as the ratio of the power spectrum within the 0.01–0.1 Hz low-frequency range to that of the whole frequency range (Zou et al., 2008). Owing to the high colinearity between ALFF and fALFF, we only included the fALFF value in the subsequent concordance calculation, as it improves specificity and sensitivity when examining regional brain activity (Zou et al., 2008; Zuo et al., 2010a; Yan et al., 2013).
(ii) ReHo: ReHo was adopted to represent the level of regional brain activity coherence. It was accepted as Kendall’s coefficient of concordance of the BOLD time course of a specific voxel with its 26 neighboring voxels’ time course (Zang et al., 2004).
(iii) GSCorr: GSCorr was considered the Pearson’s correlation coefficient between the averaged time series and time series of each voxel within the entire gray matter (Hahamy et al., 2014; Yang et al., 2017; Zhang et al., 2019). Afterward, the above GSCorr values underwent Fisher’s z-transformation to reach distribution normality.
(iv) VMHC: VMHC was adopted as the Pearson’s correlation coefficient between the time series of each voxel in one hemisphere and the time series of its symmetrical counterpart in the opposite hemisphere (Zuo et al., 2010b). Subsequently, the above VMHC values underwent Fisher’s z-transformation to reach distribution normality.
(v) DC: We computed the Pearson’s correlation coefficients between the time series of all the pairwise voxels within the entire gray matter. This correspondingly resulted in the FC matrix of the entire gray matter. DC was accepted as the sum of positive FC (defined as FC values above a threshold of 0.25) between a given voxel and the rest of the voxels (Buckner et al., 2009; Zuo et al., 2012).

Our study is an exploratory analysis and aims to examine the aberrant variability and concordance of dynamic resting-state fMRI indices (i.e., dALFF, dfALFF, dReHo, dVMHC, dGSCorr, and dDC) in patients with MDD who experienced childhood trauma. Following extensive exploratory analysis, we observed significant variability differences in dALFF and dDC. However, no significant variability difference was identified for the other metrics.

### Concordance Analysis

Concordance values were computed based on Kendall’s W coefficient. Two types of concordance indices were calculated: (i) volume-wise concordance, computed as the global level concordance index across voxels; and (ii) voxel-wise concordance, computed as the voxel-level concordance across time windows of each subject.

### Statistical Analysis

Statistical analyses were conducted using SPSS software version 19.0 (IBM Corp., Armonk, NY, United States). Demographic data, clinical scale scores, and volume-wise concordance were compared between groups using the chi-square test and one-way ANOVA with *post hoc* Bonferroni correction. To compare voxel-wise concordance and standardized SD maps between groups, one-way ANOVA with *post hoc* Bonferroni correction for multiple comparisons was conducted. Significant results are obtained from the multiple comparisons with Bonferroni correction *post hoc* tests. Family-wise error correction (FWE) was conducted with a significance threshold of *p* < 0.05 and a cluster size of > 15 voxels (Wang et al., 2014). Mean dynamic index values were extracted from brain regions showing significant intergroup differences in the voxel-wise dynamic analyses. In the multiple comparisons in regions with differences in dDC, *p* < 0.05/2 = 0.025 was accepted as significant owing to dDC analysis resulting in two significant clusters. In the multiple comparisons in regions with differences in voxel-wise concordance, *p* < 0.05/3 = 0.016 was accepted as significant (voxel-wise concordance analysis resulting in three significant clusters). Pearson’s correlation analyses were used to explore the associations of voxel-wise concordance with CTQ score in all participants [*p* < 0.05/18 = 0.0027, with Bonferroni correction of 18 being due to three clusters and 6 scales (i.e., CTQ scale and its five subscales)]. In addition, to further quantitatively compare correlation coefficients between groups, we used a regression model with group moderating the associations of dynamic indices with CTQ score (*p* < 0.05/3 = 0.016, with Bonferroni correction of 3 due to three clusters). In this study, age, gender, and education were considered as control variables.

## Results

### Demographic Data

As shown in Table 1, no significant differences were found between the patients with MDD-associated childhood trauma, patients without MDD-associated childhood trauma, and control groups with respect to demographic data, Hamilton Anxiety Rating Scale score, and Hamilton Depressive Rating Scale score. However, the CTQ score and its subscale scores significantly differed between groups.

**TABLE 1:** Demographic and clinical scale scores of MDD with childhood trauma, MDD without childhood trauma, and HC group.

	MDD with childhood trauma (*n* = 48)	MDD without childhood trauma (*n* = 30)	HC (*n* = 103)	F/t/x^2^	*p*-value
Age (years), mean ± SD	28.1 ± 6.524	29.07 ± 7.913	27.03 ± 6.591	1.185	0.308
Gender (male/female)	24/23	11/19	44/59	2.436	0.119
Educational level (years), mean ± SD	12.92 ± 3.319	13.73 ± 3.35	14.32 ± 2.598	3.778*	0.025
MDD onset age	27.9 ± 6.722	28.00 ± 7.424	26.94 ± 7.268	2.122	0.560
HAMD score	29.46 ± 8.543	29.73 ± 5.458	–	0.025	0.876
HAMA score	16.65 ± 6.849	19.7 ± 6.276	–	3.91	0.052
Mean FD (mm)	0.564 ± 0.021	0.582 ± 0.021	0.561 ± 0.017	0.141	0.869
CTQ score	55.33 ± 12.575	29.7 ± 4.535	38.09 ± 9.126	78.385**	<0.001
Emotional neglect	18.04 ± 3.984	7.43 ± 2.921	11.15 ± 4.729	66.043**	<0.001
Physical neglect	12.19 ± 3.486	5.77 ± 1.04	8.31 ± 2.927	51.169**	<0.001
Emotional abuse	11.02 ± 4.987	5.73 ± 1.165	7.06 ± 2.678	30.897**	<0.001
Physical abuse	8.06 ± 4.503	5.57 ± 1.165	6.07 ± 1.767	11.035**	<0.001
Sexual abuse	6.02 ± 2.686	5.2 ± 0.407	5.5 ± 1.065	2.754	0.066

*p < 0.05, **p < 0.01. MDD, major depressive disorder; CTQ, childhood trauma questionnaire; HC, healthy control.

### Dynamics of Resting-State Functional Magnetic Resonance Imaging Indices

**FIGURE 1:**
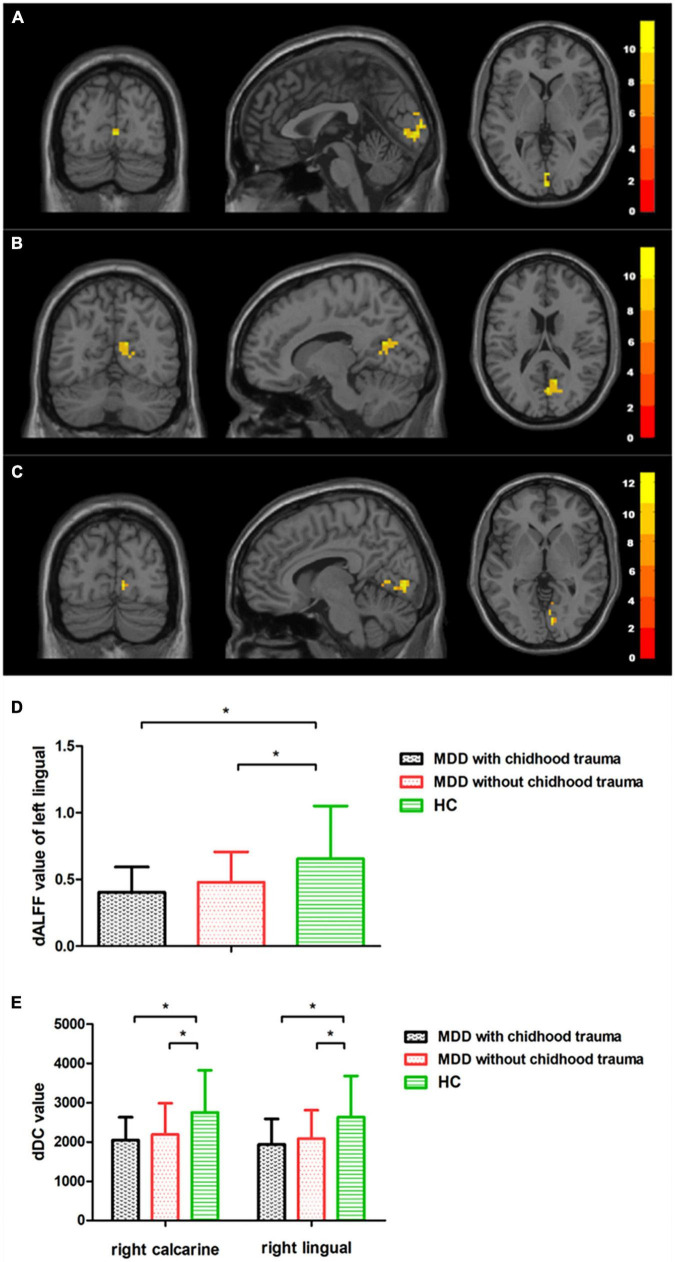
Regions with differences in dALFF and dDC between the MDD with childhood trauma, MDD without childhood trauma, and HC groups and *post hoc* analysis. Relative to HC, MDD with childhood trauma showed decreased dALFF in left lingual and decreased dDC in right calcarine and right lingual. MDD: major depressive disorder; HC: healthy control; dALFF, dynamics of amplitude of low-frequency fluctuations; dDC, dynamics of degree centrality. * means the *p*-value has reached a significant level. In the multiple comparisons in regions with differences in dDC, *p* < 0.05/1 = 0.05 was accepted as significant; In the multiple comparisons in regions with differences in dDC, *p* < 0.05/2 = 0.025 was accepted as significant.

Intergroup differences in dALFF were detected in the left lingual gyrus (Table 2 and Figure 1), whereas differences in dDC were observed in the right lingual gyrus and the right calcarine cortex (Table 2 and Figure 1). The *post hoc* testing showed that dALFF and dDC were lower in the patients with childhood trauma-associated MDD group than in the patients without childhood trauma-associated MDD and the control group (Table 3 and Figure 1). Other dynamic indices did not significantly differ between groups.

**TABLE 2:** Regions with differences in dynamic R-fMRI indices among the MDD with childhood trauma, MDD without childhood trauma, and HC groups.

Anatomical region	Peak MNI			Cluster size	*F*
	*x*	*y*	*z*		
dALFF					
Left lingual	0	−81	3	36	11.6394
dDC					
Right lingual	9	−81	0	26	12.7027
Right calcarine	12	−63	18	78	11.8277

*dALFF, dynamics of amplitude of low-frequency fluctuations; dDC, dynamics of degree centrality; MDD, major depressive disorder; HC, healthy control.*

**TABLE 3:** Multiple comparisons in regions with differences in dALFF and dDC.

Dynamic R-fMRI indices	Anatomical region	(I)	(J)	Mean difference (I-J)	*p*	95% CI	
dALFF	Left lingual	MDD with childhood trauma	MDD without childhood trauma	−0.0622708	0.413	−0.212051	0.08751
			HC	−0.2512045*	<0.001	−0.363675	−0.138734
		MDD without childhood trauma	MDD with childhood trauma	0.0622708	0.413	−0.08751	0.212051
			HC	−0.1889337*	0.006	−0.32245	−0.055417
		HC	MDD with childhood trauma	0.2512045*	<0.001	0.138734	0.363675
			MDD without childhood trauma	0.1889337*	0.006	0.055417	0.32245
dDC	Right lingual	MDD with childhood trauma	MDD without childhood trauma	−114.333	0.589	−531.23	302.56
			HC	−696.346*	<0.001	−1009.4	−383.3
		MDD without childhood trauma	MDD with childhood trauma	114.333	0.589	−302.56	531.23
			HC	−582.013*	0.002	−953.64	−210.38
		HC	MDD with childhood trauma	696.346*	<0.001	383.3	1009.4
			MDD without childhood trauma	582.013*	0.002	210.38	953.64
	Right calcarine	MDD with childhood trauma	MDD without childhood trauma	2043.499*	<0.001	1646.31	2440.69
			HC	−708.469*	<0.001	−1006.72	−410.22
		MDD without childhood trauma	MDD with childhood trauma	−2043.499*	<0.001	−2440.69	−1646.31
			HC	−2751.968*	<0.001	−3106.03	−2397.91
		HC	MDD with childhood trauma	708.469*	<0.001	410.22	1006.72
			MDD without childhood trauma	2751.968*	<0.001	2397.91	3106.03

*P_adjust_ was set as 0.05/2 = 0.025. MDD, major depressive disorder; HC, healthy control.

### Volume-Wise Concordance of Resting-State Functional Magnetic Resonance Imaging Indices

**FIGURE 2:**
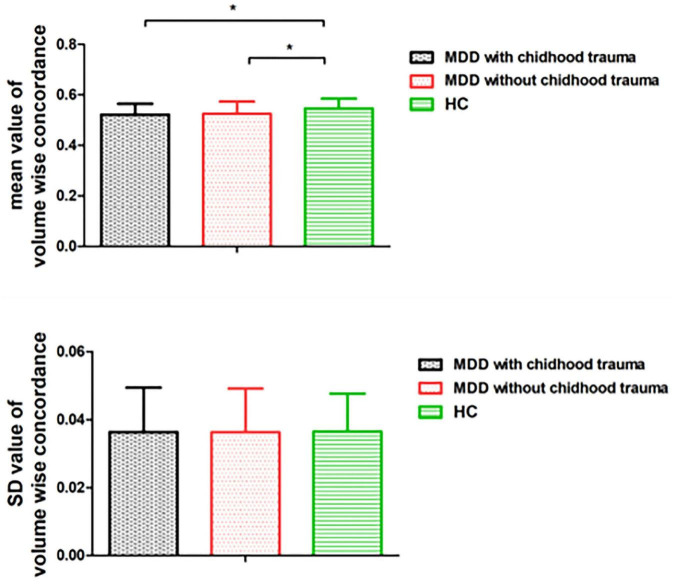
Comparison of volume wise concordance among the MDD with childhood trauma, MDD without childhood trauma, and HC groups. MDD, major depressive disorder; HC, healthy control. *p < 0.05.

Mean volume-wise concordance values significantly differed among the three groups (*p* = 0.001). The *post hoc* testing showed that mean concordance was lower in the patients with childhood trauma-associated MDD group than in the control group. SD values of volume-wise concordance did not significantly differ between the three groups (*p* = 0.996; Figure 2 and Table 4).

**TABLE 4:** Comparison of volume wise concordance among the MDD with childhood trauma, MDD without childhood trauma, and HC groups.

	MDD with childhood trauma	MDD without childhood trauma	HC	*F*	*p*
Mean	0.521 ± 0.043	0.524 ± 0.048	0.546 ± 0.038	7.068	0.001
SD	0.036 ± 0.013	0.036 ± 0.012	0.036 ± 0.011	0.004	0.996

*MDD, major depressive disorder; HC, healthy control.*

### Voxel-Wise Concordance of Resting-State Functional Magnetic Resonance Imaging Indices

**FIGURE 3:**
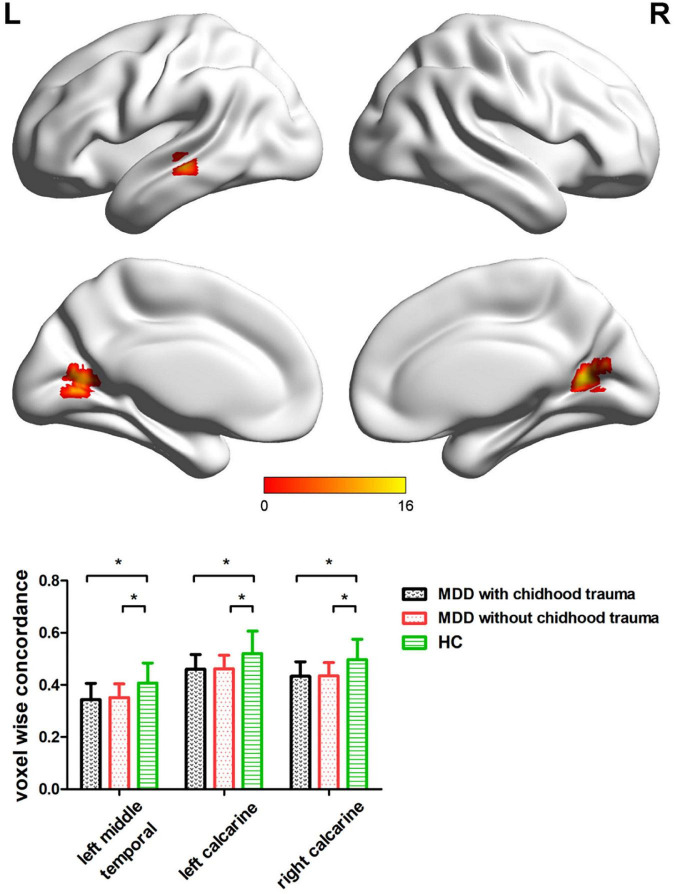
Regions with differences in the voxel wise concordance of R-fMRI indices among the MDD with childhood trauma, MDD without childhood trauma, and HC groups and *post-hoc* analysis. MDD, major depressive disorder; HC, healthy control. * means the *p*-value has reached a significant level. In the multiple comparisons in regions with differences in voxel-wise concordance, *p* < 0.05/3 = 0.016 was accepted as significant (voxel-wise concordance analysis resulting in three significant clusters).

Significant differences were observed in the left middle temporal and bilateral calcarine cortices when comparing voxel-wise concordance between the groups (Figure 3 and Table 5). Multiple comparisons showed that the concordance of these regions in the patients with childhood trauma-associated MDD group was lower than that in the other two groups (Figure 3 and Table 6).

**TABLE 5:** Regions with differences in the voxel-wise concordance of R-fMRI indices among the MDD with childhood trauma, MDD without childhood trauma, and HC groups.

Anatomical region	Peak MNI			Cluster size	*F*
	*x*	*y*	*z*		
Left middle temporal	−60	−27	−6	36	15.8445
Left calcarine	−21	−69	15	57	11.8452
Right calcarine	21	−60	9	68	15.5341

**TABLE 6:** Multiple comparisons in regions with differences in voxel-wise concordance.

Anatomical region	(I)	(J)	Mean difference (I-J)	*p*	95% CI	
Right calcarine	MDD with childhood trauma	MDD without childhood trauma	−0.001788	0.911	−0.03344	0.02986
		HC	−0.063534*	<0.001	−0.0873	−0.03977
	MDD without childhood trauma	MDD with childhood trauma	0.001788	0.911	−0.02986	0.03344
		HC	−0.061746*	<0.001	−0.08996	−0.03353
	HC	MDD with childhood trauma	0.063534*	<0.001	0.03977	0.0873
		MDD without childhood trauma	0.061746*	<0.001	0.03353	0.08996
Left calcarine	MDD with childhood trauma	MDD without childhood trauma	−0.002346	0.891	−0.03622	0.03153
		HC	−0.060463*	<0.001	−0.0859	−0.03503
	MDD without childhood trauma	MDD with childhood trauma	0.002346	0.891	−0.03153	0.03622
		HC	−0.058117*	<0.001	−0.08831	−0.02792
	HC	MDD with childhood trauma	0.060463*	<0.001	0.03503	0.0859
		MDD without childhood trauma	0.058117*	<0.001	0.02792	0.08831
Left middle temporal	MDD with CT	MDD without childhood trauma	−0.007787	0.63	−0.03959	0.02402
		HC	−0.063079*	<0.001	−0.08696	−0.0392
	MDD without childhood trauma	MDD with childhood trauma	0.007787	0.63	−0.02402	0.03959
		HC	−0.055292*	<0.001	−0.08364	−0.02694
	HC	MDD with childhood trauma	0.063079*	<0.001	0.0392	0.08696
		MDD without childhood trauma	0.055292*	<0.001	0.02694	0.08364

*P_adjust_ was set as 0.05/3 = 0.016.

*MDD, major depressive disorder; HC, healthy control.*

### Correlation Analysis and Multiple Linear Regression Analysis

**FIGURE 4:**
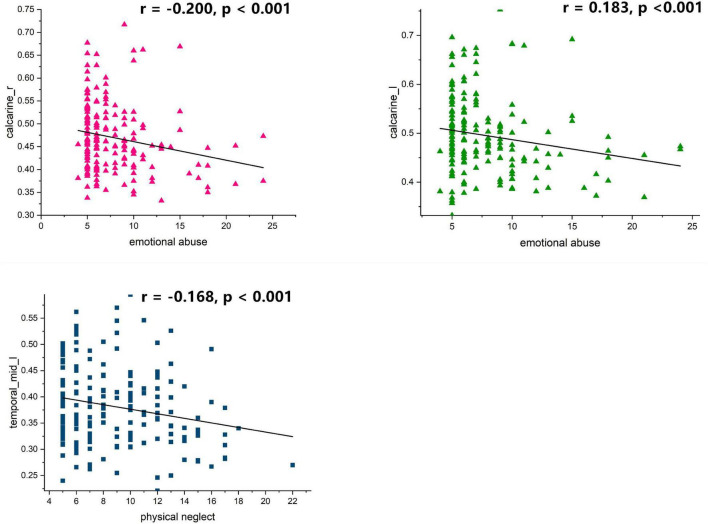
Correlation between childhood trauma history and voxel wise concordance.

We further examined the associations of dALFF and dDC with the CTQ total score. As shown in Figure 4 and Table 7, the correlation analyses revealed that childhood trauma history was negatively correlated with voxel-wise concordance in the left middle temporal (*r* = −0.166, *p* = 0.026), left calcarine (*r* = −0.160, *p* = 0.032), and right calcarine (*r* = −0.165, *p* = 0.027), respectively.

**TABLE 7:** Correlation between voxel wise concordance and childhood trauma.

	Emotional abuse	Physical abuse	Sexual abuse	Emotional neglect	Physical neglect	Total score of CTQ
Right calcarine	−0.200*	−0.221*	0.042	−0.13	−0.100	−0.165
Left calcarine	−0.183*	−0.136	−0.001	−0.149	−0.072	−0.160
Left middle temporal	−0.124	−0.160	0.108	−0.125	−0.168*	−0.166

*P_adjust_ was set as 0.05/18 = 0.0027.

Moreover, multiple linear regression analysis (Baron and Kenny, 1986) was used to investigate whether a history of childhood trauma has the same effect on dALFF and dDC in individuals with and without depression. We defined group difference (depressed or not), CTQ total score, and their interaction as independent variables; functional concordance was defined as the dependent variable; age, gender, and education were considered as nuisance covariates. To avoid multicollinearity, we performed mean centering for all the independent variables before constructing the interaction terms (Holmbeck, 1997). As shown in Table 8, further regression analyses showed an interaction of group and childhood trauma history on dALFF of the left lingual gyrus (*F* = 6.798, *p* < 0.001), dDC of the right lingual gyrus (*F* = 5.423, *p* < 0.001), and dDC of the right calcarine cortex (*F* = 5.529, *p* < 0.001).

**TABLE 8:** Multiple linear regressions analyses between childhood trauma history and dynamic indices.

Model		Unstandardized coefficients	Standardized coefficients	*t*	*p*	95% confidence interval	
		B	Beta			Lower bound	Upper bound
dALFF of left lingual gyrus	Age	0.011	0.224	3.215*	0.002	0.004	0.018
	Education	−0.018	−0.156	−2.193	0.03	−0.034	−0.002
	Gender	0.038	0.055	0.794	0.428	−0.056	0.132
	Group	0.232	0.334	4.633*	<0.001	0.133	0.33
	CTQ total score	−0.004	−0.158	−2.097	0.037	−0.008	0.000
	Group * CTQ total score	−0.0012	−0.004	−0.058	0.954	0.000	0.000
dDC of right lingual gyrus	Age	14.176	0.099	1.392	0.166	−5.92	34.272
	Education	−11.806	−0.036	−0.499	0.618	−58.513	34.901
	Gender	−123.058	−0.063	−0.896	0.371	−394.124	148.007
	Group	626.742	0.319	4.339*	<0.001	341.682	911.802
	CTQ total score	−12.595	−0.17	−2.204	0.029	−23.875	−1.315
	Group * CTQ total score	−0.011	−0.136	−1.836	0.068	−0.023	0.001
dDC of right calcarine cortex	Age	12.291	0.087	1.227	0.221	−7.472	32.053
	Education	−13.183	−0.041	−0.566	0.572	−59.114	32.748
	Gender	−104.621	−0.054	−0.775	0.44	−371.183	161.942
	Group	636.274	0.329	4.48*	<0.001	355.95	916.599
	CTQ total score	−11.588	−0.159	−2.062*	0.041	−22.68	−0.495
	Group * CTQ total score	−0.013	−0.158	−2.145	0.033	−0.025	−0.001

*P_adjust_ was set as 0.05/3 = 0.016.

## Discussion

This study adopted temporal dynamic analysis to examine aberrant variability and concordance of intrinsic brain activity in patients with childhood trauma-associated MDD. Several findings were interesting: (i) patients with childhood trauma-associated MDD exhibited lower dALFF in the left lingual gyrus and lower dDC in the right calcarine cortex as well as the right lingual gyrus relative to healthy subjects; (ii) patients with childhood trauma-associated MDD showed decreased volume-wise concordance compared with healthy controls; (iii) decreased voxel-wise concordance was observed in the left middle temporal cortex and bilateral calcarine cortices in patients with childhood trauma-associated MDD; and (iv) multiple linear regression analysis revealed that history of childhood trauma had a different impact on aberrant brain functional concordance in depressed patients and healthy subjects. However, dynamic R-fMRI index and functional concordance analyses showed no significant differences between the MDD with childhood trauma group and the MDD without childhood trauma group, which may be related to our small sample size.

Patients with childhood trauma-associated MDD had lower dALFF (mainly detected in the left lingual gyrus) and dDC (mainly detected in the right lingual gyrus and right calcarine cortex) than healthy subjects, suggesting stable but inflexible intrinsic brain activity in patients with childhood trauma-associated MDD. Previous studies have suggested that dysfunction in the lingual gyrus and calcarine cortex is closely linked to the development of MDD in patients with previous childhood trauma. Childhood trauma has been associated with impairment of emotion regulation, involving the multiprocess of emotion regulatory stages that precede and follow psychological regulatory implementation (Bonanno and Burton, 2013). Preliminary investigations that focused on the neural basis of emotion dysregulation have reported that activation in specific brain areas (including the lingual gyrus and calcarine cortex) is associated with attentional deployment, cognitive change, and response modulation (Sheppes et al., 2015).

Greater activation in the left lingual gyrus has been observed during the processing of the sadness emotion (Groves et al., 2018), suggesting that different patterns of brain activation in the lingual gyrus might be related to the underlying neural mechanisms of depression. Moreover, Daniels et al. (2012) identified a positive relationship between the history of childhood trauma and higher activation in the lingual gyrus. Evidence from temporal dynamics analysis has also revealed a key role for the lingual gyrus in processing negative emotions. In a neuroimaging study of brain dynamics in depressed patients, Zhang et al. (2021) detected significantly lower dALFF in patients with MDD relative to healthy subjects, which is in line with our findings. In a dynamic functional network connectivity analysis, Zhi et al. (2018) found that depressed patients exhibited decreased harmonic centrality values in the lingual gyrus, which was correlated with clinical symptom severity and self-cognition. In depressed patients with suicidal ideation, an aberrant dynamic functional connection between the lingual gyrus and habenula has been detected (Qiao et al., 2020). Considering the findings of prior studies as well as this study, abnormal brain activity variability in the lingual gyrus might indicate disrupted dynamic intrinsic brain activity in patients with MDD. Moreover, the lingual gyrus is widely involved in distinguishing emotional facial expressions and verbal declarative memory (Kitada et al., 2010); difficulties with these processes are common in patients with childhood trauma-associated MDD. The lingual gyrus dysfunction in patients with childhood trauma-associated MDD might reflect an increased ability to identify and encode adverse experiences in verbal declarative memory (Kitada et al., 2010). In conclusion, the alterations in variability in the lingual gyrus might be specific to the additive effects of MDD and childhood trauma history.

We detected a significant decrease in dDC in the right lingual gyrus and right calcarine cortex. Calcarine cortex dysfunction is frequently observed in patients with MDD and is closely related to depression severity. A previous study has confirmed a relationship between increased depressive symptoms and increased FC between the calcarine cortex and basolateral amygdala in veterans with MDD (McGlade et al., 2020). Additionally, a previous meta-analysis reported decreased cortical thickness in the left calcarine cortex and lingual gyrus in depressed patients compared with healthy controls (Suh et al., 2019). Similarly, decreased normalized cerebral blood flow in the right calcarine cortex in early-onset MDD patients has been observed, providing more experimental evidence for the contribution of calcarine dysfunction to the development of depression (Liao et al., 2017). More powerful evidence from FC analysis detected significantly reduced FC between the right posterior insular gyrus, calcarine cortex, and lingual gyrus in adolescents with MDD (Hu et al., 2019). Notably, childhood trauma might also cause calcarine cortex dysfunction (Luo et al., 2022). In addition to statistical analysis, dynamic analysis has also revealed a key role of abnormal calcarine variability in contributing to the negative impact of depression from the perspective of temporal dynamics. Decreased dALFF has been previously detected in the calcarine cortex (Zhang et al., 2021), which is consistent with our findings and further confirms the relationship between the development of depression and abnormal brain variability in the calcarine cortex. Similarly, by examining alterations in dfALFF, Hu, L. and colleagues identified altered variability in the calcarine cortex in depressed patients with mild cognitive impairment relative to those without mild cognitive impairment (Yu Y. et al., 2019). Furthermore, Li et al. (2021) surprisingly found that baseline functional stability in the calcarine cortex could effectively predict improvement of clinical symptoms in depressed patients. The altered variability in the calcarine cortex observed in our study may be a core neurobiological feature of MDD with childhood trauma.

Intergroup differences were found in functional voxel-wise and volume-wise concordance. Specifically, patients with childhood trauma-associated MDD showed decreased voxel-wise concordance in the left middle temporal, left calcarine, and right calcarine cortices compared with healthy controls; volume-wise concordance was also lower in patients with childhood trauma-associated MDD. The temporal gyrus is involved in language and memory function (Eichenbaum et al., 2007), whereas the calcarine cortex plays a key role in integrating “visuopsychic” and “visuosensory” processing (Ffytche and Catani, 2005). In a previous study, adults who experienced childhood trauma had increased activation in the left middle temporal gyrus and left superior frontal gyrus, indicating an association between middle temporal gyrus dysfunction and underlying neurophysiological MDD mechanisms (Heany et al., 2018). Furthermore, increased FC between the calcarine cortex and amygdala has been shown in patients with post-traumatic stress disorder, which demonstrates that the calcarine cortex plays an essential role in the processing of fear and threat cues (Morey et al., 2015). Moreover, R-fMRI metrics have been shown to have a high concordance in cortical and subcortical areas across the whole time window (Yan et al., 2017). Voxel-wise concordance might characterize the homogeneity between the various R-fMRI metrics (Lou et al., 2021). Therefore, aberrant functional concordance in the left middle temporal and left and right calcarine cortices might reflect the impaired integrative function of intrinsic brain activity.

In our multiple linear regression analyses of dynamic indices and childhood trauma history, group differences (depressed or not) significantly moderated the relationship between dALFF and dDC and childhood trauma history, indicating that childhood trauma has a significantly different impact on aberrant brain functional concordance in depressed patients and healthy subjects. This result highlights the key role of childhood trauma in mental health development and provides evidence that it is detrimental.

### Limitations and Future Directions

This study had several limitations. First, we used a cross-sectional approach, which does not examine cause and effect. Second, the sample size was small, which is probably why we could not detect a difference between MDD patients with and without childhood trauma. Future large-scale studies are warranted. Moreover, childhood trauma subtype analyses were not conducted due to the small sample size. Future studies should focus on the impact of a single subtype of childhood trauma, such as neglect or abuse. Third, childhood trauma was assessed retrospectively *via* self-report; although the CTQ is reliable and widely used, evaluation of traumatic history using an objective tool would have been preferable.

## Conclusion

Patients with childhood trauma-associated MDD demonstrated aberrant variability and concordance in intrinsic brain activity. These aberrances may be an underlying neurobiological mechanism that explains MDD from the perspective of temporal dynamics.

## Data Availability Statement

The original contributions presented in the study are included in the article/Supplementary Material, further inquiries can be directed to the corresponding author/s.

## Ethics Statement

The studies involving human participants were reviewed and approved by the Ethics Committee of the Affiliated Brain Hospital of Guangzhou Medical University. The patients/participants provided their written informed consent to participate in this study.

## Author Contributions

HP, HW, and QL designed the study and drafted the primary manuscript. HW, ZW, and QL supervised the recruitment and made statistical analyses. JC, QL, and YL took part in recruitment and data management. HP and HW made further revisions to the manuscript. All authors had read and approved the final manuscript.

## Conflict of Interest

The authors declare that the research was conducted in the absence of any commercial or financial relationships that could be construed as a potential conflict of interest.

## Publisher’s Note

All claims expressed in this article are solely those of the authors and do not necessarily represent those of their affiliated organizations, or those of the publisher, the editors and the reviewers. Any product that may be evaluated in this article, or claim that may be made by its manufacturer, is not guaranteed or endorsed by the publisher.

## Footnotes

1
www.restfmri.net/forum/DPARSF

2
http://rfmri.org/dpabi
