# Supplementary material for: Altered Variability and Concordance of Dynamic Resting-State Functional Magnetic Resonance Imaging Indices in Patients With Major Depressive Disorder and Childhood Trauma
Source: Front Neurosci. 2022 May 9;16:852799. doi: 10.3389/fnins.2022.852799 (PMC9124829; doi:10.3389/fnins.2022.852799)
Supplement: Supplementary file 1 [file Data_Sheet_1.docx]

Supplementary Material

**Supplementary tables**

**Table S1** Regions with differences in R-fMRI indices of dynamics among the MDD with childhood trauma, MDD without childhood trauma, and HC groups (window length 30TR)

|  | Anatomical region | Peak MNI | | | cluster size | F |
| --- | --- | --- | --- | --- | --- | --- |
|  |  | x | y | z |  |  |
| dALFF | Left lingual | 0 | -81 | 3 | 43 | 11.8291 |

**Table S2** Regions with differences in R-fMRI indices of dynamics among the MDD with childhood trauma, MDD without childhood trauma, and HC groups (window length 70TR)

|  | Anatomical region | Peak MNI | | | cluster size | F |
| --- | --- | --- | --- | --- | --- | --- |
|  |  | x | y | z |  |  |
| dALFF | Left cuneus | -9 | -84 | 27 | 39 | 14.9835 |
|  | Left calcarine | -18 | -66 | 6 | 9 | 9.8881 |
| dDC | Right lingual | 9 | -81 | 0 | 18 | 12.1076 |
|  | Right calcarine | 12 | -63 | 18 | 79 | 12.1158 |
|  | Right cuneus | 3 | -84 | 15 | 22 | 11.7289 |

**Table S3** Comparison of volume-wise concordance among the MDD with CT, MDD without CT, and HC groups. (window length 30TR)

|  | MDD with CT | MDD without CT | HC | F | P |
| --- | --- | --- | --- | --- | --- |
| Mean | 0.493±0.038 | 0.497±0.044 | 0.516±0.0368 | 6.687 | 0.002 |
| SD | 0.044±0.012 | 0.044±0.012 | 0.045±0.011 | 0.25 | 0.779 |

**Table S4** Comparison of volume-wise concordance among the MDD with CT, MDD without CT, and HC groups. (window length 70TR)

|  | MDD with CT | MDD without CT | HC | F | P |
| --- | --- | --- | --- | --- | --- |
| Mean | 0.542±0.479 | 0.546±0.512 | 0.569±0.396 | 7.482 | 0.001 |
| SD | 0.030±0.013 | 0.029±0.013 | 0.029±0.010 | 0.140 | 0.869 |

**Table S5** Regions with differences in the voxel-wise concordance of rs-fMRI indices among the MDD with childhood trauma, MDD without childhood trauma, and HC groups. (window length 30TR)

| Anatomical region | Peak MNI | | | cluster size | F |
| --- | --- | --- | --- | --- | --- |
|  | x | y | z |  |  |
| left middle temporal | -60 | -27 | -3 | 16 | 14.4767 |
| left lingual | -3 | -78 | 0 | 15 | 12.3451 |
| right calcarine | 21 | -63 | 9 | 17 | 13.1001 |
| right postcentral | 39 | -30 | 48 | 14 | 11.849 |

**Table S6** Regions with differences in the voxel-wise concordance of rs-fMRI indices among the MDD with childhood trauma, MDD without childhood trauma, and HC groups. (window length 70TR)

| Anatomical region | Peak MNI | | | cluster size | F |
| --- | --- | --- | --- | --- | --- |
|  | x | y | z |  |  |
| Left middle temporal | -60 | -24 | -6 | 12 | 14.517 |
| Right calcarine | 18 | -60 | 9 | 11 | 9.2036 |

**Table S7** Correlation between voxel wise concordance and childhood trauma. (window length 30TR)

|  | Emotional abuse | Physical abuse | Sexual abuse | Emotional neglect | Physical neglect | Total score of CTQ |
| --- | --- | --- | --- | --- | --- | --- |
| Right calcarine | -0.222^**^ | -0.271^**^ | -0.059 | -0.147^*^ | -0.143 | -0.234^**^ |
| Left lingual | -0.171^*^ | -0.12 | -0.048 | -0.128 | -0.114 | -0.169^*^ |
| Right postcentral | -0.123 | -0.141 | -0.053 | -0.153^*^ | -0.204^**^ | -0.195^**^ |
| Left middle temporal | -0.188^*^ | -0.16^*^ | -0.135 | -0.173^*^ | -0.226^**^ | -0.243^**^ |

^**^ Correlation is significant at the 0.01 level (2-tailed).

^*^ Correlation is significant at the 0.05 level (2-tailed).

**Table S8** Correlation between voxel wise concordance and childhood trauma. (window length 70TR)

|  | Emotional abuse | Physical abuse | Sexual abuse | Emotional neglect | Physical neglect | Total score of CTQ |
| --- | --- | --- | --- | --- | --- | --- |
| Right calcarine | -0.214^**^ | -0.212 | 0.005 | -0.153^*^ | -0.082 | -0.197^**^ |
| Left middle temporal | -0.194^**^ | -0.198 | -0.106 | -0.092 | -0.157^*^ | -0.196^**^ |

^**^ Correlation is significant at the 0.01 level (2-tailed).

^*^ Correlation is significant at the 0.05 level (2-tailed).

**Supplementary figure**


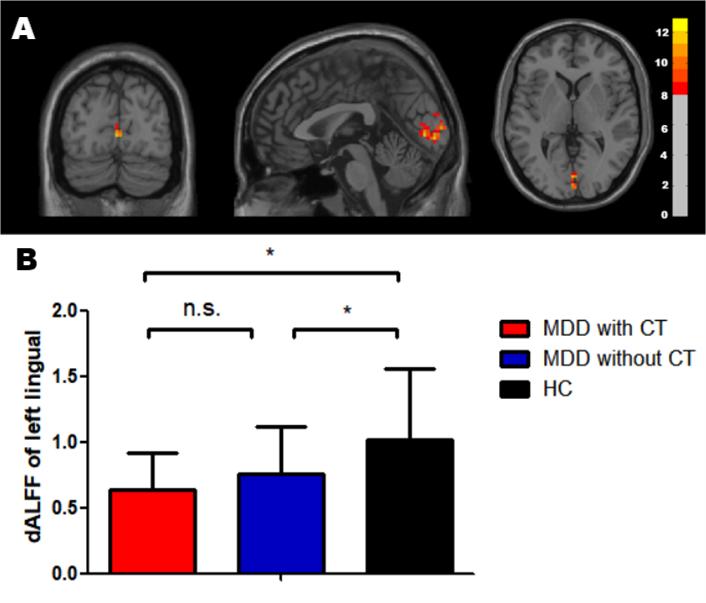


**Fig S1.** Regions with differences in dALFF and dDC between the MDD with childhood trauma, MDD without childhood trauma, and HC groups and post-hoc analysis. (window length 30TR)


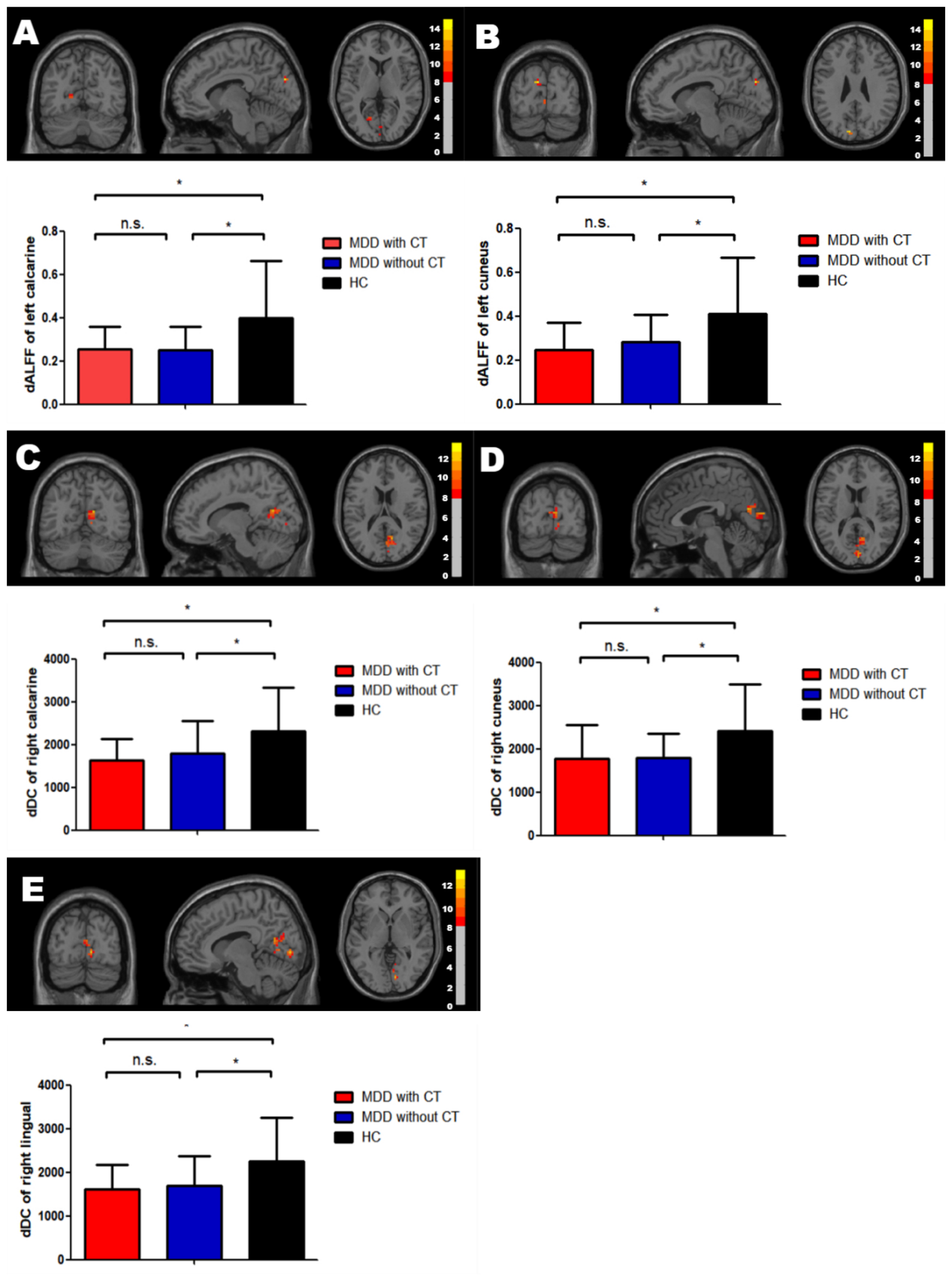


**Fig S2.** Regions with differences in dALFF and dDC between the MDD with childhood trauma, MDD without childhood trauma, and HC groups and post-hoc analysis. (window length 70TR)


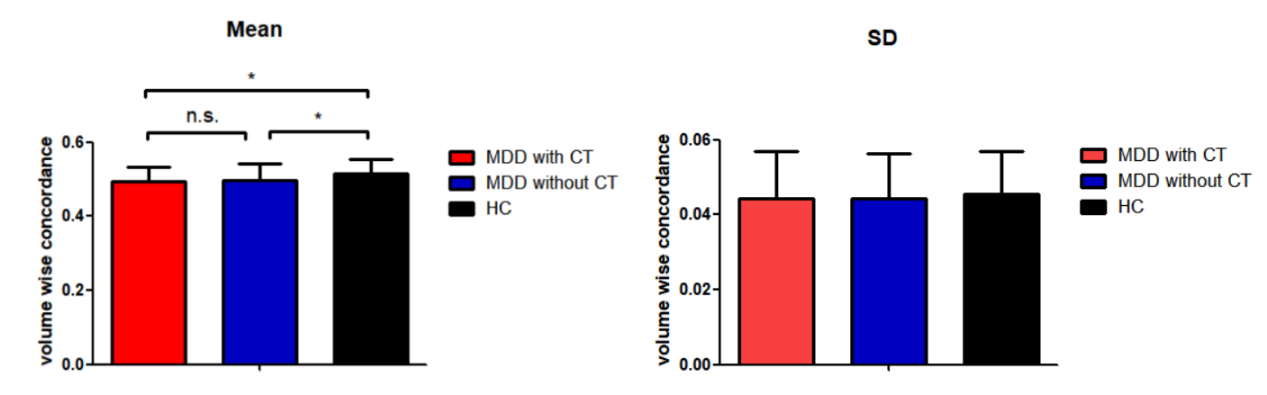


**Fig S3.** Comparison of volume-wise concordance among the MDD with childhood trauma, MDD without childhood trauma, and HC groups. (window length 30TR)


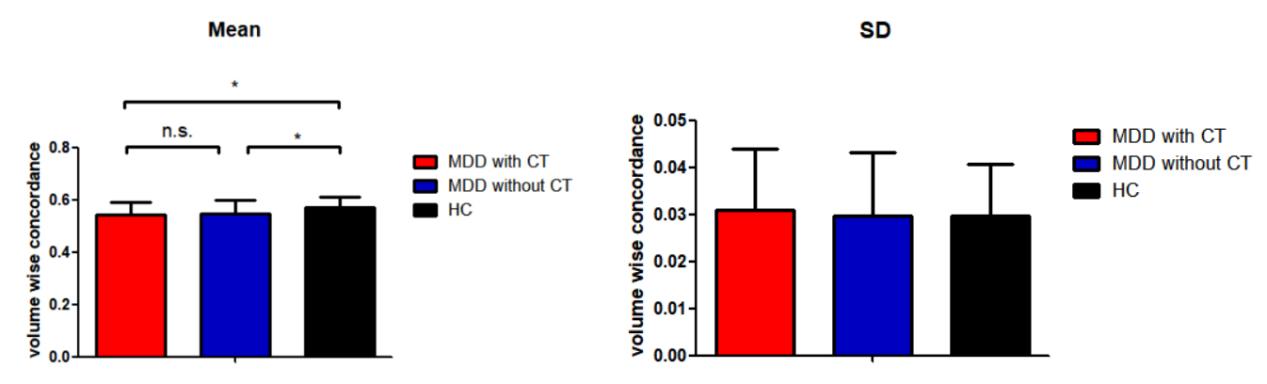


**Fig S4.** Comparison of volume-wise concordance among the MDD with childhood trauma, MDD without childhood trauma, and HC groups. (window length 70TR)


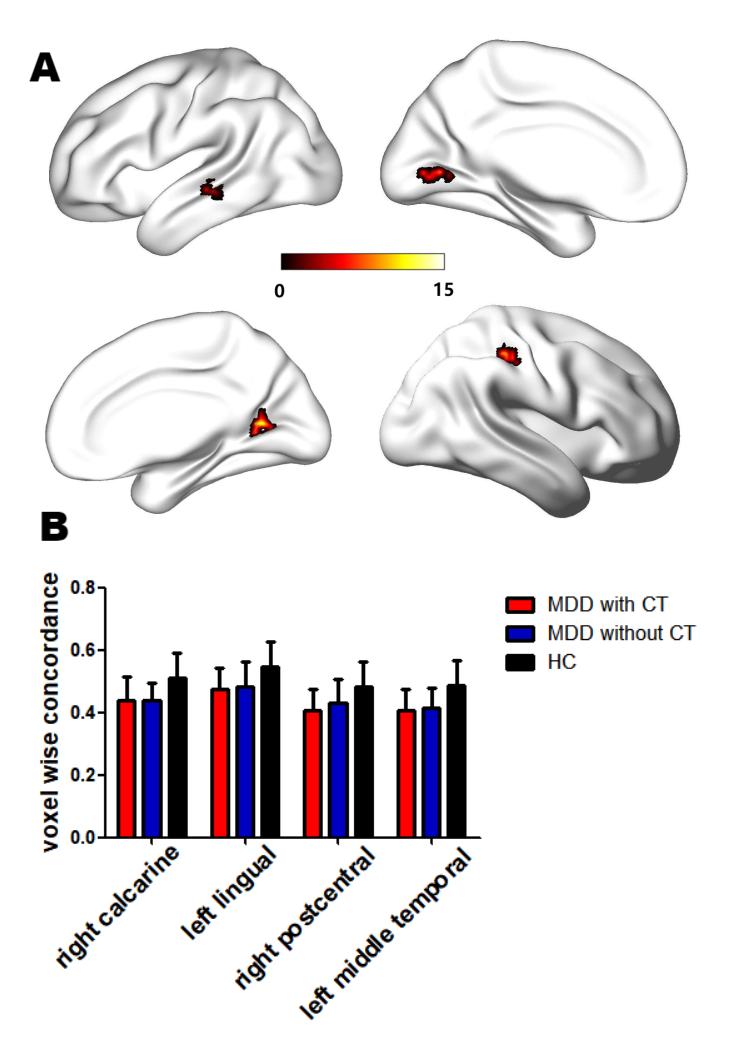


**Fig S5.** Regions with differences in the voxel wise concordance of rs-fMRI indices among the MDD with CT, MDD without CT, and HC groups and post-hoc analysis. (window length 30TR)


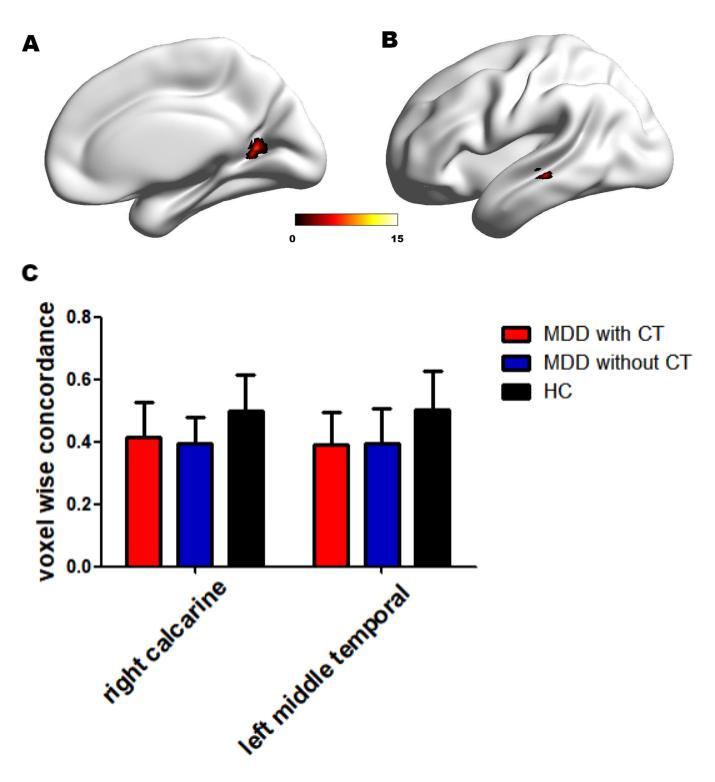


**Fig S6.** Regions with differences in the voxel wise concordance of rs-fMRI indices among the MDD with CT, MDD without CT, and HC groups and post-hoc analysis. (window length 70TR)


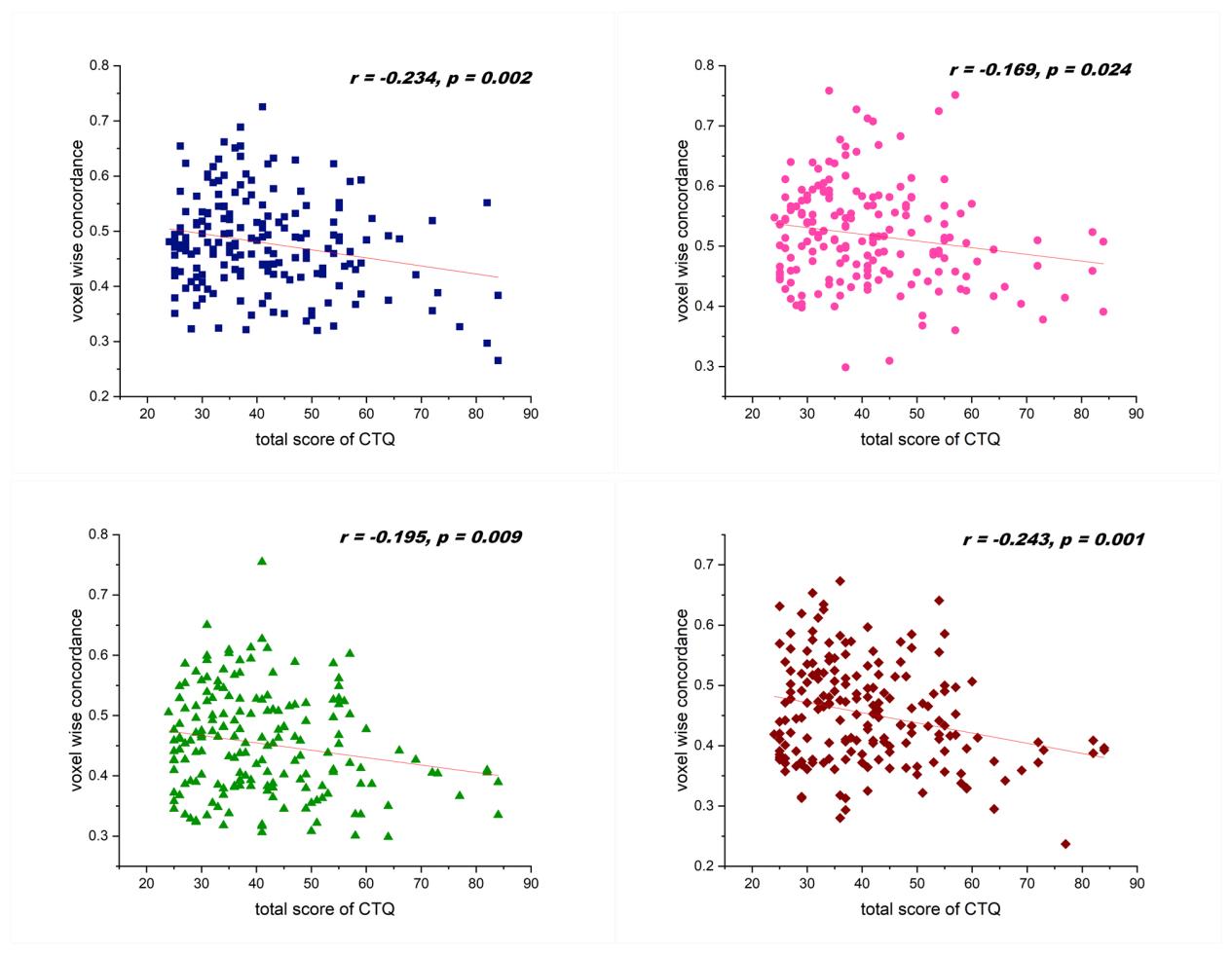


**Fig S7.** Correlation between volxel wise concordance and childhood trauma. (window length 30TR)


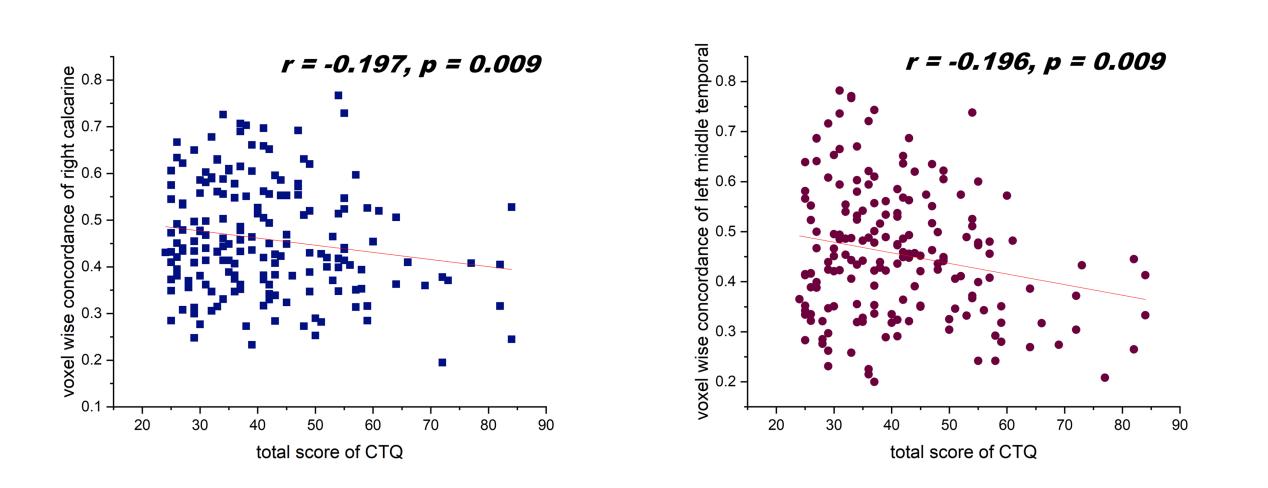


Fig S8. Correlation between volxel wise concordance and childhood trauma. (window length 70TR)
